# Continuous BP monitoring of ICU Taiwanese patients: training-phase evaluation of oCare™ BP100 according to ISO 81060-3: 2022

**DOI:** 10.3389/fmedt.2026.1681323

**Published:** 2026-07-07

**Authors:** Chao-Lun Lai, Yeh-Wen Lee, Chia-Fang Chang, Chang-Sheng Chu

**Affiliations:** 1 Department of Internal Medicine, National Taiwan University Hospital Hsin-Chu Branch, Hsin-Chu, Taiwan; 2 Department of Internal Medicine, College of Medicine, National Taiwan University, Taipei, Taiwan; 3 Taiwan Biophotonic Corporation (tBPC), Zhubei, Hsinchu County, Taiwan

**Keywords:** continuous blood pressure monitor, ISO 81060-3, non-invasive, photoplethysmogram (PPG), Type T mode

## Abstract

**Objective:**

This study aimed to conduct a training-phase evaluation of a novel continuous automated non-invasive sphygmomanometer, oCare™ BP100, in intensive care unit (ICU) Taiwanese patients according to ISO 81060-3:2022 in Type T mode. The evaluation included stability testing and blood pressure change tracking.

**Methods:**

A total of forty ICU patients were recruited from the National Taiwan University Hospital Hsin-Chu Branch. All enrolled patients had undergone unilateral radial artery catheterization in the forearm as part of routine clinical care. The catheter was connected to Philips patient monitors (Models: IntelliVue MX800 and X2) to obtain invasive blood pressure measurements for calibration purposes. Each subject underwent simultaneous monitoring with oCare™ BP100 sensors placed on the fingers of both hands. Paired measurements of the same side were further processed and followed ISO 81060-3:2022, for the defined Type T blood pressure monitor, the well-established stability method and the blood pressure change method were implemented and analyzed.

**Results:**

The present study belongs to the training phase and therefore the study was not carried out completely according to the ISO 81060-3:2022 protocol, e.g., the distribution of gender and the eligible subject in BP change test not fulfilled. For all 6 analysis periods, for the corrected standard deviations of SBP, DBP, and MAP parameter defined ISO target thresholds (A) s_corr_ ≤ 6 mmHg for each analysis period. In the Blood Pressure Change Test, at most 22 subjects the minimum requirement of 50 change points per subject, the for the change requirements were (B1) ${\bar{E}}_{50th}\leq 25\text{\%}$ and (B2) ${\bar{E}}_{85th}\leq 50\text{\%}$ error for blood pressure.

**Conclusion:**

The present study demonstrated that a training-phase evaluation of Type T mode continuous automated non-invasive sphygmomanometer repeated SBP, DBP, and MAP measurement of oCare™ BP100 in Type T mode are stable across various analysis periods and patients. oCare™ BP100 in Type T Mode can track blood pressure changes in ICU patients during different activities. Even with the absence of females in the sex ratio and an insufficient number of participants in the blood pressure change test, the exploratory study still showed training-phase results that were within ISO target thresholds, full ISO validation remains to be completed.

## 1 Introduction

Hypertension affects approximately more than 1 billion adults worldwide. Hypertensive patients undergo a healthy lifestyle and hypertensive drug use is based on hypertension device measurements (1). Traditional blood pressure measurement technologies, such as office blood pressure (OBP) and home blood pressure measurement (HBP), are limited by the time of operation and can only provide fragmented information, unable to automatically grasp day and night changes in blood pressure. Since the dynamic change in blood pressure is a challenge, the snapshot measurements provided by the current OBP or HBP are insufficient (2). Although cuff-type 24 h blood pressure monitoring can provide blood pressure data at some time points, its measurement intervals are long and interfere greatly with the user's activities and sleep. It is not suitable for long-term or real-time physiological monitoring and cannot be effectively applied to clinical needs such as pre/postoperative blood pressure monitoring, sudden blood pressure abnormalities during sleep, and detecting of blood pressure changes related to sleep disorders.

A plethysmograph is an instrument that measures changes in volume within an organ or the whole body. A photoplethysmogram (PPG) is an optically obtained plethysmogram that can be used to detect changes in blood volume in the microvascular bed of tissue. This technology had been applied in continuous blood pressure monitoring (CBPM) (3, 4), but the clinical validation of most PPG-related CBPM lack of comparison with an appropriate gold standard, such as a sphygmomanometer or invasive arterial blood pressure (5). Even the validation process was done according the state of the art (6, 7), the accuracy was mainly focused on intermittent blood pressure measurements rather than continuous blood pressure measurements (8, 9). Not to mention the issue of confirming the change test of continuous blood pressure between the CBPM and gold standard.

The investigational medical device under evaluation is oCare^™^ BP100, a prototype of non-invasive continuous blood pressure monitoring system developed by Taiwan Biophotonic Co. (tBPC). The main function is to use a finger-clip sensor to receive photoplethysmography (PPG) signals and to collect continuous blood pressure values uninterruptedly within the initialization time. By analyzing the PPG waveform characteristics and combining them with algorithms, we can achieve the ability to output blood pressure values every 30 s to meet the clinical demand for “continuous and accurate” blood pressure monitoring.

Currently, commercially available devices that are tested and validated according to the ISO 81060-3 standard only partially comply with subject recruitment and testing procedures (10, 11). In this study, according to the latest published ISO 81060-3:2022 standard requirements, non-invasive blood pressure measurements by cuff of “Philips” Patient Monitor (Model: IntelliVue MX800 & X2) at start time points were used as oCare^™^ BP100 blood pressure calibration. Invasive arterial catheter blood pressure measurements of the A-line were used as the reference invasive BPM equipment (“Philips” Patient Monitor, Model: IntelliVue MX800 & X2). The objectives of the study were to confirm whether the stability of oCare^™^ BP100 within the ISO target thresholds of calibration standard deviation ≤6 mmHg per analytical cycle; and whether the performance of oCare^™^ BP100 in changes of blood pressure was consistent with reference measurements.

## 2 Material and methods

### 2.1 Cuffless blood pressure device

**Figure 1:**
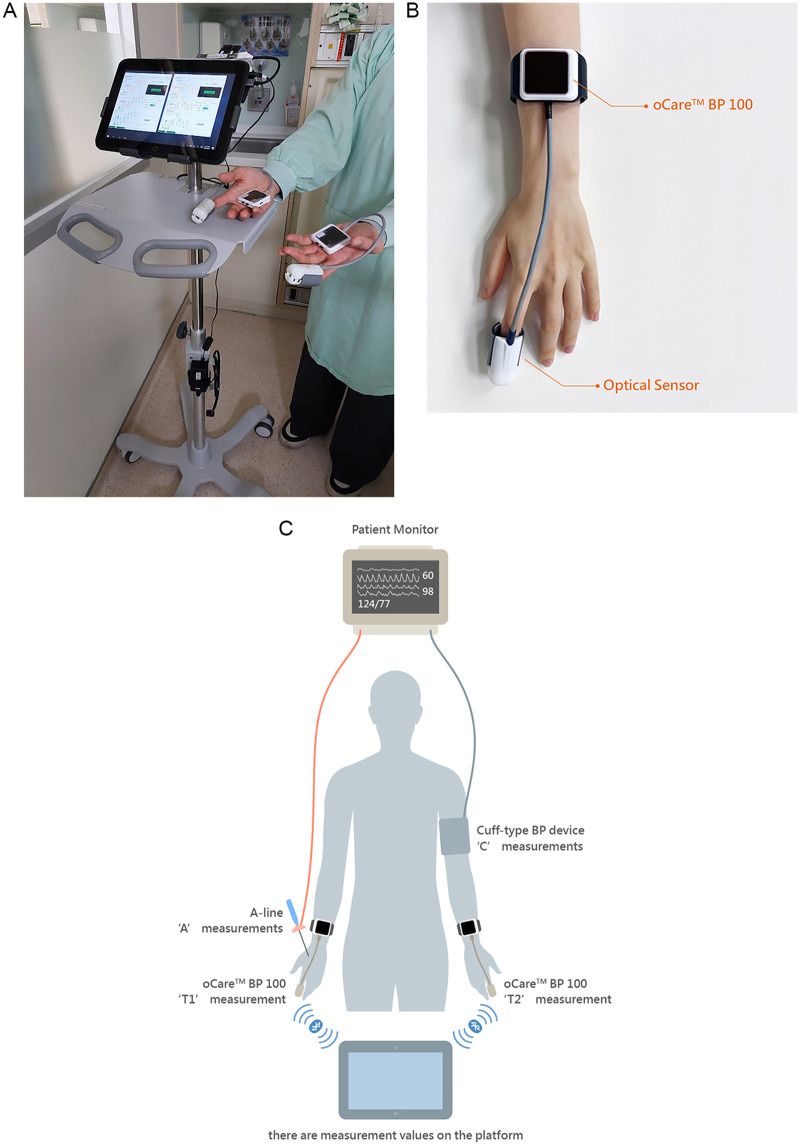
Illustration of oCare™ BP100 and reference device. **(A)** Two oCare™ BP100 and Monitor Panel. **(B)** oCare™ BP100. **(C)** Two oCare™ BP100 and Reference Device with A-Line and Cuff.

**Figure 2:**
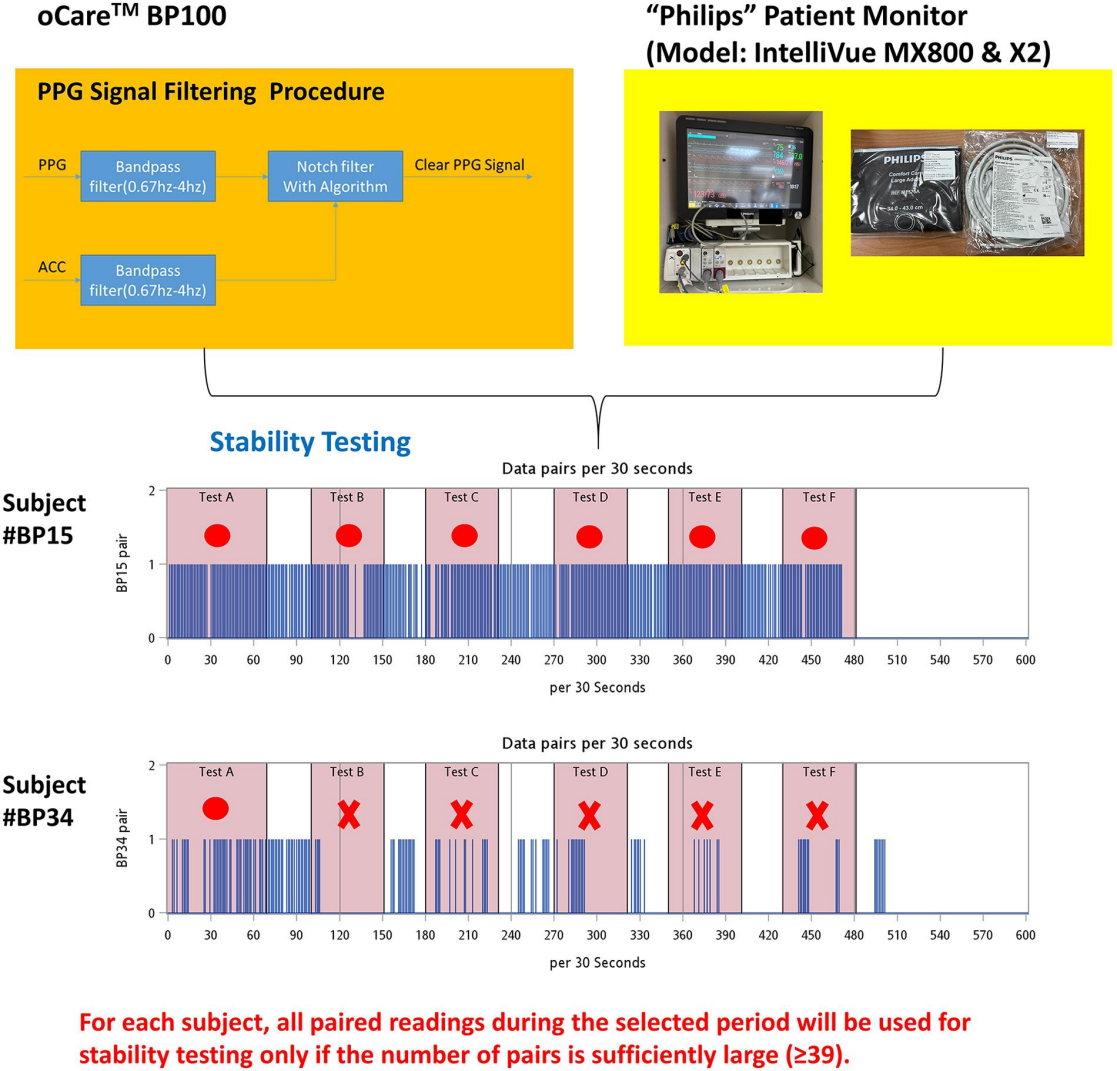
PPG signal filtering procedure of oCare™ BP100.

A prototype cuffless device developed by Taiwan Biophotonic Corporation (tBPC), oCare^™^ is a continuous blood pressure monitor made by optical detection technology. [Software Version:V3.000; Firmware Version: 3.025 (r0)]. The shell of the oCare^™^ BP100 sensor is in contact with the subject's finger, and the material is biomedical resin. The finger bed of the oCare^™^ BP100 sensor is in contact with the subject's finger and the material is high-temperature vulcanized silicone. The oCare^™^ BP100 watch strap is in contact with the wrist or hand of the subject, and the material is silicon-based thermoplastic vulcanizate. The oCare^™^ BP100 case is in contact with the subject's wrist or hand and is made of polycarbonate. Those who are allergic to the above materials should not use this device. To achieve optical cuffless continuous blood pressure measurement technology, the algorithm developed by tBPC implements an integrated approach utilizing multiple advanced techniques. This algorithm not only analyzes blood pressure through detailed photoplethysmography (PPG) waveform features but also incorporates PPG waveform decomposition technology to significantly enhance measurement accuracy. Furthermore, the system employs multi-wavelength detection technology, effectively reducing external interference factors and ensuring measurement data stability and reliability. The synergistic application of these technologies provides a non-invasive continuous blood pressure monitoring solution with considerable potential for clinical applications (Figures 1, 2).

### 2.2 Reference blood pressure

The reference BP includes 4 h continuous invasive blood pressure (ABP) monitoring provided by the “Philips” Patient Monitor (Model: IntelliVue MX800 & X2). The same cuff model Philips M1574A or M1575A cuff was only used for Time calibration at both the start and end points of the BP measurements (*N*IBP) (12). The cuff was attached to the arm opposite line A and removed during the 4-hour measurement period. Attach the NIBP tubing to the NBP connector on the X2 module. On the Philips IntelliVue MX800 and X2, ABP and NIBP are handled through the X2 module, which serves as the acquisition device for the MX800 host (13).

### 2.3 Recruitment

This study was conducted in the National Taiwan University Hospital (NTUH) Hsin-Chu Branch. A total of 40 intensive care unit (ICU) patients were recruited with the inclusion criteria as follows:

Inclusion Criteria.

- Age ≥ 22 years at the time of informed consent
- Already receiving radial artery cannulation (A-line) for blood pressure monitoring based on clinical indication
- Ability to understand the written informed consent form and willingness to participate, as indicated by signed informed consent from the patient or legal representative

Exclusion Criteria.

- Pregnancy
- Use of intra-aortic balloon pump
- Presence of arm or finger injuries
- History of vascular surgery involving the upper extremities or contraindications to local treatment in the upper extremities
- Arterial line placement in the brachial artery
- Determined unsuitable for study participation based on investigator judgment (e.g., poor compliance, impaired mental status, unstable health condition)
- Refusal by the participant or legal representative to sign the informed consent form

Subjects who met the inclusion criteria and signed the informed consent form were formally enrolled in the study protocol. If the measurements were incomplete or the waveform did not meet the normal pattern, the data were invalid for downstream analysis. Common types of poor-quality PPG signals observed in this study include: (1) high-frequency noise, primarily resulting from improper attachment of the fingertip sensor to the patient's finger or from motion-induced vibrations at the detection site during monitoring, leading to significant oscillatory artifacts in the PPG waveform; (2) waveform distortion, often caused by external compression of the sensor, which in turn applies pressure to the measurement site, resulting in abnormal deformation of the waveform morphology; and (3) marked attenuation or near absence of the AC component in the PPG signal, where the pulsatile waveform is significantly reduced or flat, compromising the identification of systolic peaks and other morphological features. Subjects underwent follow-up within 7 days post-trial to evaluate adverse events (AEs). The study concluded thereafter.

### 2.4 Procedure

1. The researcher confirms that the subject has the A-line embedding needle for radial artery blood drawing, and the “Philips” patient monitor (Model: IntelliVue MX800 & X2) is on (named “A” measurements). The side with A-line referred to as side A.
2. Turn on the notebook computer that can receive two units of oCare™ BP100.
3. For Side A, the researcher assists the subject in wearing one unit of oCare™ BP100 (named “T1” measurements), turns it on, and confirms that there are signals on the platform.
4. Attach the blood pressure cuff of “Philips” Patient Monitor to the arm on the other side B, and connect to the NIBP slot of “Philips” Patient Monitor (Model: IntelliVue MX800 & X2) (named “C” measurements).
5. For Side B, the researcher assists the subject in wearing another unit of oCare™ BP100 (named “T2” measurement), turns on it and confirms that there are measurement values on the platform.
6. Start the cuff-type blood pressure measurements on the arm of side B. This is the starting point of the test.
7. Start the 4 h measurement on the reference medical device and investigational medical device.
8. Start the cuff-type blood pressure measurements on the arm of side B. This is the end point of the test.
9. Stop measurements, and shut down the two investigational medical devices. And remove the two investigational medical devices and the cuff of the “Philips” Patient Monitor.
10. The researchers confirm the storage and recording of measured values on the platform.
11. End of the test.
12. Physicians continued patient care in accordance with the pre-established treatment plan.
13. Researchers conducted follow-up visits with subjects within seven days following the completion of monitoring.

### 2.5 Ethics

The protocol has been approved by the 175th meeting of Research Ethics Committee B of the National Taiwan University Hospital on Oct 20, 2023. The committee is organized under, and operates in accordance with, the Good Clinical Practice guidelines and governmental laws and regulations. The duration of this approval is one year (from Oct 20, 2023 to Oct 19, 2024). NTUH-REC No.: 202309072DSB.

### 2.6 Trial registration

The project has been registered in ClinicalTrials.gov (ID: NCT06815614) https://clinicaltrials.gov/study/NCT06815614.

### 2.7 Algorithm

The device utilizes green, red, and infrared wavelengths at a 200 Hz sampling rate. This multiwavelength approach enhances signal quality against variations in skin tone, motion, and perfusion by capturing surface features (green) and deep vascular data (red/IR); specifically, certain IR bands assist in calibration due to stable hemoglobin absorption. The algorithm applies band-pass filtering and leverages accelerometer (ACC) data to eliminate motion-induced noise from PPG signals. Calibration is a single-point procedure that maps 30 s of extracted PPG features to NIBP measurements. Artifact detection relies on ACC-derived motion thresholds and a 90% similarity requirement between adjacent PPG pulses. If valid pulses comprise less than 60% of the 30 s window, the blood pressure calculation is suspended. For re-initialization, the system continuously scans and resumes calculation once PPG features meet these quality standards within a subsequent 30-second period. This framework ensures technical transparency and adherence to ISO 81060-3 standards in complex environments.

## 3 Standard requirements and methods

All statistical analyses were performed in accordance with the ISO 81060-3:2022 standard for clinical investigation of continuous automated non-invasive sphygmomanometers. And, all analyses were implemented in SAS 9.4+. This analysis included both stability [Clause 5.2 in (10)] and blood pressure changes [Clause 5.3 in (10)], and complied with the detailed procedures defined in Annex D in (10) (13) (Tables 1A, 1B).

1. Stability (Clause 5.2 in (10)):
  1.1 The initialization time of the investigational medical device is four hours, according to the requirements of ISO 81060-3: 2022, the minimum ≧ six test periods, each one at least 15-minutes long. It is estimated that the subjects will wear the investigational medical device for four hours to record all test data and then capture six test periods. Each test period is divided into analysis periods, each analysis period contains 29 repeated measurement values for each subject.
  1.2 According to each subject i, take the average value of the difference between all reference readings and blood pressure determinations from the first analyzable period of the first test period as the ith subject. It can be the representative value of individual differences in all analysis periods of all test periods.
    $$o_{i}=\frac{1}{r}\sum_{\;j=1}^{r}\left(P_{sut_{j}}-P_{ref_{j}}\right)$$ (1)
  1.3 For each pair of data, *j*, of each subject *i,* within each analysis period from each test period, calculate the offset corrected difference of the paired values, $x_{\;j,T}$ using Formula (2) considering the subject-specific offset oi as calculated in (1).
    $$x_{\;j,T}=P_{sut_{j}}-P_{ref_{j}}-o_{i}$$ (2)
  1.4 For each analysis period of each test period calculate over the totality, the corrected experimental standard deviation, $S_{corr}$ were calculated (Table 1A).
2. Blood Pressure Changes [Clause 5.3 in (10)]:
  2.1 Set the change evaluation interval to 30 min
    2.1.1 Divide the data recorded from the reference invasive blood pressure monitoring equipment into non-overlapping segments of approximately equal duration.
    2.1.2 Match the end time of each segment to the initial time the respective output value provided by the continuous automated non-invasive sphygmomanometer as closely as possible.
  2.2 Pair Blood Pressure measurements
    2.2.1. If the segment contains more than one reference reading, average the reference readings of each segment.
    2.2.2. Match the end time of each segment to the initial time the respective output value provided by the continuous automated non-invasive sphygmomanometer as closely as possible.Pair the representative value at the time zone point with the output value of the investigational medical device and mark it as a time scale.
    2.2.3. Each paired blood pressure value is the starting point of a blood pressure change analysis, and the end point is calculated from the starting point $t_{start}$, and the length of the two does not exceed the change evaluation interval.
    2.2.4. If re-initialization occurs, the blood pressure change will be discarded and not included.
    2.2.5. Calculate the change in blood pressure for each device.
    2.2.6. When the blood pressure change for the investigational medical device or the blood pressure change for the reference device meets the corresponding requirements,
      2.2.6.1. SBP change absolute value ≥15 mmHg,
      2.2.6.2. DBP change absolute value ≥10 mmHg,
      2.2.6.3. MAP change absolute value ≥12 mmHg,

**Table 1A:** Notations for stability test.

Notation	Definition
*n*	The total number of paired measurements of all subjects
*i*	The *i*th subject
*j*	The *j*th paired measurement
k	The total number of subjects
r	For each subject, the number of repeated paired measurements
$m_{i}$	For the *i*th subject, the number of repeated paired measurements
$x_{i}$	For the *i*th subject, the mean of the differences
${\bar{x}}_{i}$	The means of the difference of all paired values of the *i*th subject
$x_{j}$	The differences of the the *j*th paired measurement
$\bar{x}=\frac{1}{n}\overset{n}{\underset{\;j=1}{\sum }}\;x_{j}$	The overall mean of the differences of all paired values of all subjects
$x_{\;j,T}$	The offset corrected difference of the paired values
$o_{i}=\frac{1}{r}\overset{r}{\underset{\;j=1}{\sum }}\;(P_{sut_{j}}-P_{ref_{j}})$	For the *i*th subject, the subject-specific offset from the first analysis period
$S_{corr}=\sqrt{\frac{\mu_{SB}-\mu_{SW}}{\;f_{BA}}+\mu_{SW}}$	The corrected experimental standard deviation
$f_{BA}=\frac{n^{2}-\sum m_{i}^{2}}{(k-1)n}$	A Bland-Altman factor
$\mu_{SB}=\frac{1}{k-1}\overset{k}{\underset{i=1}{\sum }}\;m_{i}({\bar{x}}_{i}-\bar{x})\sigma_{i}^{2}=\frac{1}{k-1}\overset{k}{\underset{i=1}{\sum }}\;r({\bar{x}}_{i}-\bar{x})\sigma_{i}^{2}$	The mean of the squares between subjects
$\mu_{SW}=\frac{1}{n-k}\overset{k}{\underset{i=1}{\sum }}\;(m_{i}-1)\sigma_{i}^{2}=\frac{1}{n-k}\overset{k}{\underset{i=1}{\sum }}\;(r-1)\sigma_{i}^{2}$	The mean of the squares within a subject
$\sigma_{i}^{2}$	The variance of the differences of the paired values of the *i*th individual subject

**Table 1B:** Notations for blood pressure change test.

Notation	Definition
Δ*P_ref_*	the change in BP for the reference device, IntelliVue MX800 & X2
Δ*P_sut_*	the change in BP for the oCare™ BP100
$E_{\;percent}=\frac{\left|\Delta P_{sut}-\Delta P_{ref}\right|}{\max(\left|\Delta P_{sut}|,|\Delta P_{ref}\right|)}\times 100\text{\%}$	For each subject, the percentile of absolute error
$E_{50th}$	For each subject, the 50th percentile of absolute error
$E_{85th}$	For each subject, the 85th percentile of absolute error
${\bar{E}}_{50th}$	The averaged of $E_{50th}$ values over all subjects
${\bar{E}}_{85th}$	The averaged of $E_{85th}$ values over all subjects

then calculate the absolute error, $E_{\;percent}$, according to Formula [3].

$$E_{\;percent}=\frac{\left|\Delta P_{sut}-\Delta P_{ref}\right|}{\max(\left|\Delta P_{sut}|,|\Delta P_{ref}\right|)}\times 100\text{\%}$$ (3)

     Δ*P_ref_*: the change in BP for the reference sphygmomanometer.

Δ*P_sut_*: the change in BP for the oCare™ BP100.

  2.2.7. After pooling all $E_{\;percent}$ values of each subject, calculate the 50th percentile of all the $E_{\;percent}$ values per subject, $E_{50th}$.
  2.2.8. After pooling all $E_{\;percent}$ values of each subject, calculate the 85th percentile of all the $E_{\;percent}$ values per subject, $E_{85th}$.
  2.2.9. Averaging the calculated 50th overall subjects, obtain ${\bar{E}}_{50th}$. Averaging the calculated 85th overall subjects, obtain ${\bar{E}}_{85th}$.

2.3 Confirm the following endpoints.
  2.3.1. ${\bar{E}}_{50th}\leq 25\text{\%}$: After averaging the calculated 50th percentiles over all subjects, this average shall be less than or equal to 25%.
  2.3.2. ${\bar{E}}_{85th}\leq 50\text{\%}$: After averaging the calculated 85th percentiles over all subjects, this average shall be less than or equal to 50%.

The ISO target thresholds for the Stability Test is defined as (A): the corrected standard deviation s_corr_ ≤ 6 mmHg. For the Blood Pressure Changes, the criteria are (B1): the average 50th percentile of relative errors ≤25%, and (B2): the average 85th percentile of relative errors ≤50%.

## 4 Results

The results were evaluated according to the ISO target thresholds defined in the Methods section: (A) for stability, and (B1) and (B2) for blood pressure changes.

### 4.1 Participant selection, general characteristics and blood pressure distribution

During 2023/10/01∼2024/09/30, this study was conducted in the NTUH Hsin-Chu Branch. A total of 40 ICU participants were initially selected and included in the training phase according to ISO 81060-3:2022, including 29 men and 11 women participants, who had a mean age of 67.2 ± 12.6 years, with a range of 45–96 years (Table 2).

**Table 2:** Demographic characteristic of subjects.

Characteristics	*N*/mean ± SD	%/Min-Max
Sample Size	40	
Sex		
Male	29	72.5
Female	11	27.5
Age		
40–49	3	7.5
50–59	8	20.0
60–69	13	32.5
70–79	9	22.5
> = 80	7	17.5
Age (cont.)	67.2 ± 12.6	45–96

### 4.2 Stability test

Since the initialization period was set 1.5–5 h, there were 6 analysis periods were required for each subject. There must be at least 15 min between two analysis periods. Ideal (pair) readings are required 29 readings in each analysis period, subjects without ≥29 readings were invalid in the calculation for the selected segment. Table 3 lists the valid pair readings and Subject Number used for each analysis period.

**Table 3:** Number of readings and sample size of subjects used in stability test.

Analysis Period	A	B	C	D	E	F
	(0, 2,040)	(3,000, 4,500)	(5,400, 6,900)	(8,100, 9,600)	(10,500, 12,000)	(12,900, 14,400)
Order in Minutes	0–34	50–75	90–115	135–160	175–200	215–240
Order of Readings	1–68	100–150	180–230	270–320	350–400	430–480
BP01	32	40	37	34	25	39
BP05	41	47	37	34	30	45
BP06	63	49	45	10	0	14
BP07	67	42	51	49	47	45
BP09	48	14	10	19	7	4
BP13	54	33	7	22	33	0
BP14	64	48	45	2	51	23
BP15	66	40	45	48	49	40
BP16	66	46	42	45	0	0
BP19	64	46	44	50	43	50
BP20	66	46	48	45	49	21
BP21	45	33	47	9	0	0
BP23	43	43	0	0	0	0
BP24	58	51	47	9	29	25
BP27	57	41	49	43	39	0
BP28	37	34	7	27	21	11
BP31	29	34	33	44	47	47
BP32	63	31	48	35	41	45
BP34	39	4	13	12	7	11
BP35	51	41	45	39	27	28
BP37	57	49	0	0	0	0
BP38	65	43	49	0	31	36
BP40	46	33	44	48	50	50
Number of Subjects	23	21	17	12	13	9

Table 4 shows that for the corrected standard deviations of SBP, DBP, and MAP parameter within the ISO target thresholds (A) s_corr_ ≤ 6 mmHg for each analysis period. The corrected standard deviation listed for Period A–F:

1. SBP: 3.15, 3.83, 3.67, 3.99, 4.13, 4.29;
2. DBP: 1.84, 2.35, 2.25, 2.23, 2.85, 2.65;
3. MAP: 2.17, 2.54, 2.48, 2.69, 2,81, 3.12.

**Table 4:** Stability test analysis.

Period Name	A			B			C			D			E			F		
(in Secs)	(0, 2,040)			(3,000, 4,500)			(5,400, 6,900)			(8,100, 9,600)			(10,500, 12,000)			(12,900, 14,400)		
Number of Subjects	23			21			17			12			13			9		
Male Number (%)	18 (78.26%)			17 (80.95%)			13 (76.47%)			9 (75%)			10 (76.92%)			9 (66.67%)		
Female Number (%)	5 (21.74%)			4 (19.05%)			4 (23.53%)			3 (25%)			3 (23.08)			6 (33.33%)		
Period in Minutes	0–34			50–75			90–115			135–160			175–200			215–240		
	SBP	DBP	MAP	SBP	DBP	MAP	SBP	DBP	MAP	SBP	DBP	MAP	SBP	DBP	MAP	SBP	DBP	MAP
X	0.00	0.00	0.00	0.08	0.05	0.04	0.42	−0.10	−0.04	0.33	−0.35	−0.26	−0.16	−0.04	−0.17	−0.72	−0.48	−0.74
μ_SB_	0.00	0.00	0.00	106.99	49.95	53.36	58.10	60.85	44.10	192.92	52.85	71.19	108.79	78.61	57.82	191.51	133.51	139.81
μ_SW_	10.11	3.45	4.82	12.38	4.44	5.27	12.45	3.80	5.29	11.66	3.84	5.71	15.37	6.81	6.98	14.97	4.48	7.11
*f*_BA_	52.97	52.97	52.97	41.38	41.38	41.38	44.44	44.44	44.44	42.77	42.77	42.77	55.11	55.11	55.11	50.34	50.34	50.34
***S*_corr_**	**3**.**15**	**1**.**84**	**2**.**17**	**3**.**83**	**2**.**35**	**2**.**54**	**3**.**67**	**2**.**25**	**2**.**48**	**3**.**99**	**2**.**23**	**2**.**69**	**4**.**13**	**2**.**85**	**2**.**81**	**4**.**29**	**2**.**65**	**3**.**12**

Bold values indicate results that comply with the ISO target thresholds (A) (*S*_corr_ ≤ 6 mmHg).

The above values represent that repeated measurement of SBP, DBP, and MAP of the investigational medical device are stable, even the gender ratio variation and the number drop-off from Period A to Period F. The corrected experimental standard deviation S_corr_, are related to the total number of participants and the number of paired measurements. In Table 4, the fewer the participants, the higher the S_corr_.

### 4.3 Blood pressure changes

**Figure 3:**
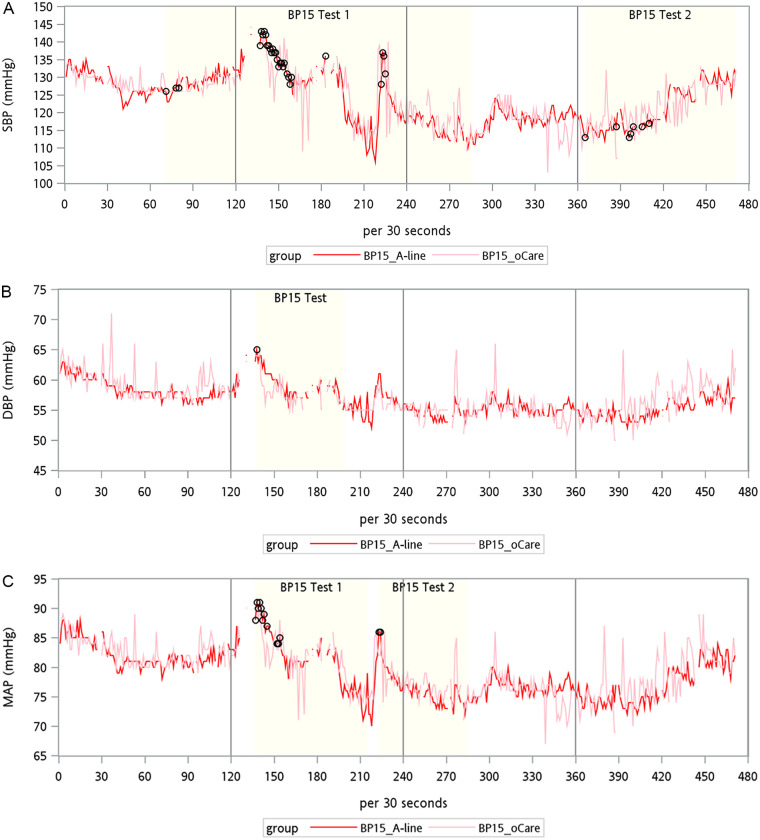
A case having rapid BP change in the change evaluation interval of 30 min. (Subject ID: BP15) **(A)** SBP. **(B)** DBP. **(C)** MAP.

For each blood pressure parameter to be investigated, the criteria (B1) and (B2) are analyzed. For example, the valid reading of blood pressure in SBP, DBP, and MAP was demonstrated in the circle symbols for Subject (ID: BP15), (Figure 3). For each subject, the valid periods varied due to SBP, DBP, and MAP. Invalid BP changes were excluded from the BP Change test analysis. Table 5 lists the analysis period and the invalid number of readings used in the blood pressure change test. Table 6 presents the ${\bar{E}}_{50th}$ of SBP, DBP, and MAP are 11.40%, 9.93%, and 10.08%; and ${\bar{E}}_{85th}$ of SBP, DBP, and MAP are 27.81%, 24.84%, and 26.31%. The ISO target thresholds for the monitoring of Blood Pressure Change Test (B1) ${\bar{E}}_{50th}\leq 25\text{\%}$ and (B2) ${\bar{E}}_{85th}\leq 50\text{\%}$ are fulfilled, the secondary hypothesis is established: the efficacy of the investigational medical device and the reference medical device in blood pressure change tests are consistent. The number of participants in the blood pressure change test was 22, 15, and 16 for SBP, DBP, and MAP, respectively. In generally, DBP changes are relatively mild and usually remain within a smaller range. In this test, its fluctuation range is inherently smaller than SBP and MAP. Therefore, the percentile of absolute errors *E_50th_* and *E*_85th_ were directly related to the absolute error of change difference between oCare™ BP100 and the reference device in hemodynamic measurements of systolic and diastolic blood pressure. Gender distribution is not a direct influencing factor for Stability Test nor BP Change Test.

**Table 5:** Invalid BP change reading pairs for all subjects (%).

Subject No.	SBP (*N* = 22)		DBP (*N* = 15)		MAP (*N* = 16)	
	Analysis time interval (secs)	Pairs	Analysis time interval (secs)	Pairs	Analysis time interval (secs)	Pairs
BP01	930–2,760	62	-	-	-	-
	2,910–6,390	117				
BP05	10,260–15,630	177	-	-	9,570–12,390	95
BP06	-	-	1,200–3,000	61	-	-
BP07	2,460–6,150	168	2,460–4,440	28	2,460–6,090	122
	10,350–12,210				10,410–12,210	61
BP08	2,880–7,890	168	-	-	-	-
BP10	5,220–14,910	299	6,750–12,000	176	5,220–14,280	298
BP13	120–4,050	132	120–4,050	132	-	-
	4,620–10,950	212	4,620–10,920	212		
BP14	150–6,510	213	-	-	1,110–4,590	117
	9,150–15,240	165				
BP15	2,130–8,550	215	4,140–5,940	61	4,140–8,520	139
	10,950–14,100	106				
BP16	1,620–9,690	270	570–6,780	208	3,330–5,130	61
BP19	-	-	5,370–7,170	61	-	-
			8,850–10,650	61		
BP20	330–2,160	62	2,820–5,820	102	2,820–5,820	101
	2,700–5,820	105	11,640–15,720	137	11,640–15,720	137
	11,640–15,840	141				
BP21	570–6,630	178	750–4,050	111	570–4,050	117
BP24	2,100–7,200	171	690–4,290	121	690–4,290	121
	12,480–16,410	132	11,760–15,360	121	11,760–15,840	137
BP25	11,340–15,420	135	-	-	-	-
BP26	9,030–1,710	177	10,830–14,370	93	9,030–14,370	179
			15,090–16,890	56	14,760–16,890	72
BP27	750–4,350	121	-	-	8,730–10,650	65
	6,240–10,800	153				
BP28	180–4,200	135	-	-	-	-
	8,160–11,670	118				
BP31	13,230–15,030	61	13,230–15,060	62	13,230–15,090	63
BP32	990–5,400	148	1,020–9,750	209	1,020–5,400	147
	10,350–12,420	70				
BP33	6,660–10,260	121	-	-	-	-
BP34	180–3,120	99	300–2,820	85	300–2,910	88
	4,830–326	166			4,830–9,780	166
BP38	690–5,850	173	-	-	690–4,170	117
	10,950–14,940	134				
BP40	60–5,400	127	60–2,250	74	60–5,250	174-
	8,520–15,390	230	10,740–14,280	119	9,060–15,390	212

**Table 6:** BP change analysis for E_50_ and E_85_ of all subjects (%).

BP	SBP		DBP		MAP	
Number of Subjects	22		15		16	
Male Number (%)	14 (63.64%)		12 (80%)		11 (68.75%)	
Female Number (%)	8 (36.36%)		3 (20%)		5 (31.25%)	
Subject No.	*E_50th_*	*E_85th_*	*E_50th_*	*E_85th_*	*E_50th_*	*E_85th_*
BP01	20.83	42.11	-	-	-	-
BP05	18.75	37.50	-	-	9.09	27.27
BP06	-	-	9.09	27.27	-	-
BP07	5.71	22.86	7.14	14.29	7.69	19.23
BP08	10.71	28.57	-	-	-	-
BP10	13.79	34.48	12.50	25.00	9.09	27.27
BP13	7.14	21.43	4.76	19.23	-	-
BP14	9.76	24.00	-	-	8.33	25.00
BP15	12.90	31.82	20.00	30.00	13.33	33.33
BP16	8.33	16.67	5.56	16.67	16.67	25.00
BP19	-	-	12.50	36.36	-	-
BP20	9.52	25.00	10.34	31.25	8.57	27.78
BP21	6.45	19.35	5.88	17.65	9.52	14.29
BP23	-	-	-	-	-	-
BP24	11.11	28.57	3.85	11.54	4.76	13.64
BP25	13.33	30.00	-	-	-	-
BP26	7.27	16.36	7.69	21.74	5.26	18.42
BP27	11.11	29.63	-	-	18.75	37.50
BP28	6.45	16.67	-	-	-	-
BP31	17.65	41.18	5.88	23.53	10.53	26.32
BP32	8.00	24.00	13.04	30.43	9.09	21.21
BP33	15.79	31.58	-	-	-	-
BP34	9.38	25.00	18.18	36.36	15.38	46.15
BP35	-	-	-	-	-	-
BP37	-	-	-	-	-	-
BP38	12.00	28.00	-	-	11.76	23.53
BP40	14.81	37.04	12.5	31.25	15.00	35.00
Mean	${\bar{E}}_{50th}=11.40$	${\bar{E}}_{85th}=27.81$	${\bar{E}}_{50th}=9.93$	${\bar{E}}_{85th}=24.84$	${\bar{E}}_{50th}=10.80$	${\bar{E}}_{85th}=26.31$

### 4.4 AE/serious adverse event (SAE)

All subjects were tracked within 7 days, none of AE/SAE was reported during the follow-up within 7 days. Two subjects died from pneumonia following study completion. These SAEs were reported and assessed as unrelated to the investigational device or the trial.

## 5 Discussion

In current published studies, rare commercial devices claim to meet the criteria of ISO 81060-3 (10). The comparisons of the proposed prototype oCare™ BP100 and related devices were detailed in Table 7. Hove *et al*. (10) presented that the DBP of healthy participants achieving the criteria of BP change test but not in SBP which were higher than the limit for systolic blood pressure (56% vs. ≤50%) and for all parameters for the 50th percentile (32%–39% vs. ≤25%). Furthermore, the importance of the study design, as specified in ISO 81060-3, is that an invasive reference is required, i.e., an invasive blood pressure monitoring device is inserted in all subjects, which was not present in their recruited healthy participants (10). Boretsky *et al*. (11) conducted a pediatric study to validate Vitalstream™, which is a medical device approved by the United States Food and Drug Administration for use in adults. Since the pediatric trial were addressed in children ages 2–17 undergoing major surgeries during hemodynamically stable periods, the validation of Blood Pressure Changes was lacking and the Vitalstream™ in Type A or Type T mode blood pressure monitoring was unclear. Khayat *et al*. (14) briefed the ambulatory BP monitoring (ABPM) passing the BP change test, even only 27 participants used which not compliance the requirement for ≥30 subjects. Detailed comparison in Table 7.

**Table 7:** Checklist for ISO 81060-3:2022 standard requirements.

Sections	Standard Requirements	Proposed Study	Boretsky (2025)	Hove (2024)	Khayat (2024)
—	Device	oCare™ BP100	Vitalstream™	A prototype photoplethysmography-based cuffless device	a novel sensor to track transient BP changes
4.2	General	Type T	Unknown (Type A?)	Unknown (Type A?)	Unknown (Type T?)
4.3.1	Reference invasive BP equipment	PASS; IntelliVue MX800 & X2	PASS; Datex Ohmeda S/5 Collect system	N.C. Human Nano or Nexfin	PASS; Undisclosed A-LINE Device
4.3.2.1	Number	PASS; Number of repeated times *r* = 29 times.	PASS; Number of Repeated times *r* = 29.	PASS; 38 heathy subjects	Limited; 27 subjects
	A clinical investigation shall consist of repeated measurements performed on test subjects. The number of repeated measurements per subject shall be determined according to the procedure in 4.5.3. The number of subjects shall be determined according to the procedure in 4.5.3.	The minimum number of subjects is 30.	31 consecutive patients aged 2–17.		
4.3.2.2	Gender distribution	N.C.	Pass;	Pass;	Unknown
	At least 30% of the subjects shall be male. At least 30% of the subjects shall be female.	Male: 72.5%	Male: 35.5%	Male: 42.1%	
		Female: 27.5%	Female: 64.5%	Female: 57.9%	
4.3.2.3.2	Age distribution for aged greater than 12 years	PASS	N.C.	N.C.	Unknown
	40% shall be at least 50 years of age; 25% shall be at least 60 years of age; and 10% shall be at least 70 years of age.	50 + yr: 92.5%	50 + yr: 0%	50 + yr: 13.2%	
		60 + yr: 62.5%	60 + yr: 0%	60 + yr: 5.3%	
		70 + yr: 40%	70 + yr: 0%	70 + yr: 0%	
4.3.2.3.3	Age distribution for aged between 1 and 12 years	Not Applicable	Pass;	Not Applicable	Not Applicable
			For aged 2–12		
4.3.3	Blood Pressure Distribution (Only for Type A)	Exempt for Type T	Exempt for aged less than 12 year.	Unknown	Unknown
			But unknown for aged 12–17		
5.1	Type A: Method for the accuracy of blood pressure determination	Exempt for Type T	Pass;	PASS	Unknown
			Bias SBP:−3.79; DBP: 1.72; MAP: 2.41 s_corr_ SBP: 9.74; DBP: 8.45; MAP: 8.57.	Bias SBP: 0.3; DBP: 0.04; MAP: 0.8. s_corr_ SBP: 8.7; DBP: 6.6; MAP:7.0.	
	Bias within or equal to ±6 s_corr_ ≤ 10 mmHg				
5.2	Method for Stability (≥30 subjects, 6 periods)	Limited; Only 15–22 subjects were included.	Unknown	Limited; Only 11 subjects were included.	Unknown
	Type A:				
	Bias within or equal to ±6; and s_corr_ ≤ 10 mmHg for each analysis period				
		s_corr_ ≤ 6 for 6 periods SBP: 3.15, 3.83, 3.67, 3.99, 4.13, 4.29; DBP: 1.84, 2.35, 2.25, 2.23, 2.85, 2.65; MAP: 2.17, 2.54, 2.48, 2.69, 2,81, 3.12.		Bias SBP: 0.3; DBP: 0.04; MAP: 0.8. s_corr_ SBP: 8.7; DBP: 6.6; MAP:7.0.	
	Type T: s_corr_ ≤ 6 mmHg for each analysis period				
5.3	Type A/Type T: Method for blood pressure changes	PASS;	Unknown	PASS	PASS
		SBP:		SBP:	(B1) 23.8% (B2) 42%
	(B1) ${\bar{E}}_{50th}\leq 25\text{\%}$ and (B2) ${\bar{E}}_{85th}\leq 50\text{\%}$	(B1) 11.40%, (B2) 27.81%,		(B1) 39%, (B2) 56%,	
		DBP:		DBP:	
		(B1) 9.93%; (B2) 24.84%		(B1) 32%; (B2) 48%	
		MAP:		MAP:	
		(B1) 10.08%, (B2) 26.31%		(B1) 33%, (B2) 47%.	

This study is the first PPG training-phase evaluation in the Type T protocol of ISO 81060-3:2022 using invasive reference measurements in a complex ICU environment. The investigational device, oCare™ BP 100, showed promising ability to meet the stability and blood pressure change criteria in a high-acuity setting. The high-frequency, every-30-second BP output meets the need for near-continuous monitoring and may contribute to improved patient safety and outcome detecting. Besides, its use of non-invasive finger-clip PPG sensing may enhance patient comfort and facilitate continuous data acquisition with minimal disruption to clinical procedures.

However, the study has some limitations. While the age distribution strictly complies with the requirements of ISO 81060-3:2022, the gender distribution did not fully comply (female participants accounted for only 27.5%, slightly below the 30% requirement). In addition, due to the prototype stage of the oCare™ BP100 sensor, readings with fewer than 29 measurements per analysis period were excluded from the Stability Test. The number of participants in the blood pressure change test was 22, 15, and 16 for SBP, DBP, and MAP, respectively. This exploratory result suggests that the change-tracking cohort falls below typical participant requirements. This selection bias or survivorship bias might limit generalizability to the most unstable ICU patients.

In future studies, further validation in accordance with the full ISO 81060-3:2022 protocol—including expanded subject demographics and testing across varied clinical scenarios—is necessary. Future evaluations involving patients with cardiovascular instability, during surgery, or in ambulatory settings will be essential to confirm the device's broader applicability and to support regulatory submission and clinical adoption.

## 6 Conclusion

This training-phase study demonstrated that the oCare™ BP100, a PPG-based non-invasive continuous blood pressure monitor, within the required ISO thresholds for stability and blood pressure changes under ISO 81060-3:2022 Type T mode in an ICU population. Stability Test were demonstrated with the correlated standard deviation less than 6 mmHg in SBP, DBP, and MAP for each analysis period. And, the Blood Pressure Changes Test was shown to be consistent with oCare™ BP100 and the reference device (“Philips” Patient Monitor, Model: IntelliVue MX800 & X2). These results support the potential of oCare™ BP100 to provide continuous and accurate hemodynamic monitoring in critical care environments. Furthermore, oCare™ BP100 needs to be validated in the downstream testing-phase per ISO 81060-3:2022 Type T mode before it can be launched on the market.

## Data Availability

The original contributions presented in the study are included in the article/Supplementary material, further inquiries can be directed to the corresponding author/s.
